# Prices Still Rising

**Published:** 1920-02-28

**Authors:** 


**THE PUBLIC WELL-BEING**—*(continued)*.

### Prices Still Rising.

The official figures of the rise in the cost of living for January have not yet been disclosed, but the *Times* understands they will show that the advance, as compared with July 1914, is now 130 per cent. The increase at the end of December stood at 125 per cent.

According to index-numbers published in the *Statist* wholesale prices during January showed a greater advance than had been recorded in any one month since the Armistice. The *Statist* figures are based on forty-five commodities, the average price of which for the eleven years 1866 to 1877 is taken as being equal to 100. The figure in June 1914 stood at 81.2; in December 1919 it was 235.2; and at the end of last month it had jumped to 245.3. This reckoning suggests that the increase in the general price-level since June 1914 has been 202 per cent., to which foodstuffs have contributed 173.7 per cent. and materials 220.7 per cent.

No immediate alleviation of the position can be reasonably expected. If some food prices are likely to decline others will rise, particularly if the American exchange continues to be against his country. It is practically certain that the price of Government butter will shortly be raised to 3s. a pound. Exporting countries on the Continent are advancing their rates, and if the butter is to come here the price demanded must be paid. In view of the heavy bread subsidy, the Government cannot even consider the possibility of subsidising any other article of food and the retail price, therefore, must be in keeping with the rate at which the butter is acquired.

Sugar prices, too, may have to be increased, and even then there is little prospect of a return to the 8-oz. ration before May. Rice is another article likely to be dearer. Potatoes are also to cost more, though if prices advance materially it may be thought advisable to reimpose control.

With regard to groceries generally, the grocers are growing discontented with the rate of profit allowed on controlled goods in face of expenses which are continually rising. Milk, eggs, and fish may fall in price in the coming weeks. New-laid eggs are already down to 4d. each.

Some striking comparisons between family budgets in 1914 and 1920 are published in the *Manchester Guardian*. The following table provides an illustration of the weekly expenditure of a family of six persons—three of them children—on groceries, bread, and milk :—

| | 1914. | | 1920. | |
|---|---|---|---|---|
| | s. | d. | s. | d. |
| Bread ... ... | 3 | 4 | 6 | 0 |
| Oatmeal ... | 1 | 4 | 3 | 8 |
| Flour ... ... | 1 | 0 | 1 | 8 |
| Margarine ... | 2 | 0 | 4 | 6 |
| Tea ... ... | 0 | 11 | 1 | 7 |
| Sugar ... ... | 0 | 7 | 2 | 8 |
| Cheese ... ... | 0 | 8 | 1 | 6 |
| Macaroni ... | 0 | 6 | 0 | 10½ |
| Rice ... ... | 0 | 6 | 0 | 8 |
| Sago ... ... | 0 | 7 | 1 | 3 |
| Eggs ... ... | 0 | 6 | 2 | 3 |
| Syrup ... ... | 0 | 6½ | 1 | 11 |
| Potatoes ... | 2 | 4 | 7 | 0 |
| Lentils ... | 0 | 8 | 0 | 11 |
| Onions ... ... | 0 | 3 | 1 | 3 |
| Milk ... ... | 2 | 4 | 6 | 5 |
| Total ...... | 18 | 0½ | 44 | 1½ |

**THE PUBLIC WELL-BEING**—(*continued*).

Another table shows the expenditure of a middle-class household consisting of husband and wife in 1914 and last year :—

| | 1914. £ | s. | d. | 1919. £ | s. | d. |
|---|---|---|---|---|---|---|
| Rent ... ... ... ... | 32 | 0 | 0 | 32 | 0 | 0 |
| House duty ... ... ... | 0 | 7 | 6 | 0 | 7 | 6 |
| Rates .. ... ... ... ... | 8 | 8 | 5 | 8 | 8 | 5 |
| Water ... ... ... ... | 2 | 18 | 0 | 2 | 18 | 0 |
| Gas ... ... ... ... ... ... | 7 | 0 | 0 | 10 | 0 | 0 |
| Insurance (personal) ... ... | 25 | 0 | 0 | 25 | 0 | 0 |
| ,, Burglary, fire, etc. ... | 1 | 0 | 0 | 1 | 0 | 0 |
| Coal ... ... ... ... ... | 4 | 10 | 0 | 10 | 0 | 0 |
| Dress ... ... ... ... ... | 20 | 0 | 0 | 50 | 0 | 0 |
| Domestic help ... ... ... | 11 | 10 | 0 | 23 | 0 | 0 |
| Railway contract ... ... | 4 | 16 | 0 | 5 | 5 | 8 |
| Holidays ... ... ... ... | 10 | 0 | 0 | 25 | 0 | 0 |
| Housekeeping ... .. ... | 60 | 0 | 0 | 143 | 0 | 0 |
| Medical, stationery, etc. ... | 4 | 12 | 0 | 10 | 0 | 0 |
| Sundries ... ... ... ... | 6 | 0 | 0 | 13 | 0 | 0 |
| | 138 | 1 | 11 | 358 | 19 | 7 |

The list makes no provision for meals in town, amusements, tobacco, presents, charities, renewals to furniture, or for increased insurance, which is really essential if adequate cover is to be made for enhanced prices on everything. The proportionate increase where there are children is greater still because of the advanced cost in education.

